# Challenges and solutions in transitioning to animal-free standards: a comprehensive analysis of components in human cell-based developmental neurotoxicity assays

**DOI:** 10.3389/ftox.2026.1800157

**Published:** 2026-05-12

**Authors:** Julia Vanessa Spänle, Lisa Maria Haiber, Bettina Seeger

**Affiliations:** 1 Research Group Food Toxicology and Alternatives/Complementary Methods to Animal Experiments, Institute for Food Quality and Food Safety, University of Veterinary Medicine Hannover, Foundation, Hannover, Germany; 2 Department of Pharmacology, Toxicology and Pharmacy, University of Veterinary Medicine Hannover, Foundation, Hannover, Germany; 3 Center for Systems Neuroscience (ZSN), Hannover, Germany

**Keywords:** chemical defined cell culture systems, developmental neurotoxicity assays, human cell-based testing, regulatory toxicology, xeno-free cell culture

## Abstract

The replacement, reduction, and refinement (3Rs) of animal experiments is a central objective in modern toxicology. Human cell-based *in vitro* assays have become key tools to implement these principles by providing mechanistically driven and human-relevant New Approach Methodologies (NAMs) for toxicity testing. However, even in approaches that avoid the use of living animals, many protocols still rely on animal-derived cell culture components beyond fetal bovine serum (FBS), such as bovine serum albumin (BSA) in supplements, extracellular matrix (ECM) preparations such as Matrigel, and animal-sourced antibodies, which may introduce variability. This review provides a targeted materials analysis of human cell-based assays within the Developmental Neurotoxicity *in vitro* Battery (DNT-IVB), examining basal media, supplements, ECM, growth factors, and antibodies. While serum-free media are widely implemented, animal-derived components remain in use, particularly in supplements, ECM, and immunodetection workflows. Guidance documents, including OECD Good *in vitro* Method Practices (GIVIMP), or Good Cell Culture Practice (GCCP), promote replacing undefined components such as FBS, but ECM and BSA are less explicitly addressed. Recommendations from the European Union Reference Laboratory for Alternatives to Animal Testing (EURL ECVAM) encourage the use of animal-free antibodies where feasible. We propose a two-pronged strategy: (1) New protocols incorporate animal-free design from inception; (2) established DNT-IVB assays evaluate component reduction where feasible, balancing validation requirements with practicality. Manufacturers can contribute through standardized labeling (“serum-free,” “animal-free”, “xeno-free,” “chemically defined”) and expanded animal-free product availability. Updated GCCP/GIVIMP guidance could explicitly address BSA and ECM as sources of variability alongside serum. By addressing persistent animal-derived reagents, this reagents-focused review advances animal-free DNT-IVB implementation and supports broader 3Rs objectives by providing actionable strategies for animal-free cell culture in human-relevant NAMs.

## 1 Introduction

Traditionally, regulatory authorities have relied heavily on animal studies to assess chemical or drug safety, including developmental toxicity. In the EU and Norway in 2022, 96,807 of 499,931 animals (19.36%) were used for regulatory developmental toxicity testing, representing the highest proportion among all toxicity endpoints (European Commission, 2024) (Table 1). This substantial animal use underscores both the critical importance of these endpoints the capacity limitations of current approaches, addressed through EU Directive 2010/63/EU’s 3Rs framework and regulatory demands for human-relevant testing (Fritsche et al., 2018).

**TABLE 1:** Regulatory uses for toxicity testing on animals in the EU and Norway in 2022.

Regulatory uses: Toxicity	Number of uses	Proportion
Developmental toxicity	96,807	19.36%
Ecotoxicity	86,306	17.26%
Repeated dose toxicity	67,349	13.47%
Reproductive toxicity	62,606	12.52%
Kinetics	46,250	9.25%
Acute and sub-acute	38,972	7.80%
Skin sensitization	33,029	6.61%
Pharmaco-dynamics (incl. Safety pharmacology)	32,043	6.41%
Safety testing in food and feed area	10,775	2.16%
Target animal safety	8,343	1.67%
Genotoxicity	4,587	0.92%
Other toxicity/safety testing	4,297	0.86%
Carcinogenicity	2,970	0.59%
Skin irritation/corrosion	2,793	0.56%
Neurotoxicity	1,489	0.30%
Phototoxicity	600	0.12%
Eye irritation/corrosion	383	0.08%
Combined end-points	332	0.07%
Total	499,931	100.00%

Developmental neurotoxicity (DNT) testing is particularly critical, as chemical-induced disruptions in neurogenesis, synaptogenesis, migration, or network formation during vulnerable developmental windows can cause lifelong neurological deficits (Rice and Barone, 2000; Grandjean and Landrigan, 2014). The DNT *in vitro* Battery (DNT-IVB), a suite of 17 cell-based assays targeting these key processes, is nearing regulatory implementation for chemical risk assessment (Bal-Price et al., 2018b; Fritsche et al., 2018; Masjosthusmann et al., 2020; EFSA, 2021; Sachana et al., 2021a; Sachana et al., 2021b; Carstens et al., 2022; Blum et al., 2023; OECD, 2023; Smirnova et al., 2024). The U.S. Environmental Protection Agency (EPA), European Food Safety Authority (EFSA) and the Organization for Economic Co-operation and Development (OECD) endorse its integration in risk assessment with OECD recommendations providing a tiered framework for its regulatory use (EFSA PPR Panel (EFSA Panel on Plant Protection Products and their Residues), OECD, 2023).

Despite substantial progress toward human-relevant *in vitro* models, a key opportunity remains to further minimize animal-derived components. Protocol optimization has successfully reduced or eliminated fetal bovine serum (FBS) in line with Good Cell Culture Practice (GCCP) recommendations (Coecke et al., 2005; Eskes et al., 2017; Pamies et al., 2017; Pamies et al., 2018; Tigges et al., 2021; Pamies et al., 2022), other animal-derived components remain prevalent and are often insufficiently documented. Directive 2010/63/EU’s classical 3Rs primarily address procedures performed on living animals and form the basis of current regulatory requirements. In contrast, modern 3Rs interpretations in New Approach Methodologies (NAMs) increasingly extend the 3Rs concept to animal-derived materials, advocating fully animal-free systems even in the absence of explicit regulatory obligations (Oredsson et al., 2019; Rosolowski et al., 2025). Bovine serum albumin (BSA) in cell culture media supplements or immunofluorescence protocols, animal-derived extracellular matrix (ECM) like Matrigel, or animal-sourced antibodies persist in protocols and commercial formulations.

This review evaluates the animal-derived, xeno-free, and chemically defined components employed in human cell-based DNT-IVB assays (definition of terms in Table 2). We identify persistent animal-derived materials despite FBS elimination, evaluate viable alternatives (basal media, supplements, ECM, growth factors, antibodies), and address implementation barriers. While broader challenges (regulatory validation, economics, comprehensive performance metrics) lie outside this reagents-focused scope, DNT-IVB exemplifies NAMs transition strategies.

**TABLE 2:** Terminology for cell culture components.

Label	Origin	Composition known?	Gray areas	References
Serum-free (SF)	Free of animal/Human serum	May be defined or undefined	May still contain animal-derived or human-derived components such as BSA, HSA	Jayme and Smith (2000), Chase et al. (2012)
Animal-free or animal-component-free (ACF)	Free of animal-derived components, (often extended to both non-human and human)	May be defined or undefined	Depending on definition including human-derived components; recombinant proteins may be acceptable, human-derived, chemically undefined proteins, may introduce variability	Jayme and Smith (2000), Rafnsdóttir et al. (2023), Mogilever et al. (2025)
Xeno-free (XF)	Proposed to describe fully animal-free technologies avoiding live animals and animal-derived components, may include human components (plasma, serum, platelet lysate, proteins)	May be defined or undefined	Safety/regulatory term in clinical use to avoid zoonotic pathogens and non-human antigens while accepting same species (human) materials; recombinant proteins which might be produced in non-human expression systems (e.g., yeast) may or may not be considered “xeno-free”” depending on how definition is applied	Chase et al. (2012), Rosolowski et al. (2025)
Chemically defined (CD)	Typically, protein-free, but not always mandated by the term alone Can contain recombinant growth factors, when purified, molecularly- defined and added at known concentrations	All components and concentrations known	Proprietary mixtures might contain trace components where labeling may not reflect full compositional transparency	Keenan et al. (2006), Huang et al. (2025)

## 2 The shift from animal testing to *in vitro* models in DNT testing

Current DNT testing protocols, such as the OECD Test Guideline (TG) 426, require pre- and postnatal exposure of animals to chemicals, followed by extensive evaluation of physical and behavioral outcomes (OECD, 2007). This approach is resource-intensive: A single DNT study conducted according to OECD TG 426 costs approximately $1.4 million, uses around 1,000 rat pups, and takes around 2 years per chemical tested (Smirnova et al., 2024). Consequently, only around 200 chemicals have been systematically assessed for DNT *in vivo* studies by the US EPA or within the OECD TG, severely limiting capacity for the thousands of chemicals in commerce (Martin et al., 2022).

Human cell-derived *in vitro* assays address these limitations by reducing time, costs, and animal use while enabling high-throughput screening. Critically, they provide human-relevant data, a key advantage given documented species differences in chemical sensitivity. Harrill et al. (2011) demonstrated that human neurons showed greater sensitivity to neurite outgrowth inhibitors than rat neurons, highlighting toxicodynamic differences between species. Similarly, Baumann et al. (2016) found that while both human and rat neural progenitor cell spheroids correctly classified nine test chemicals for DNT potential, they differed in sensitivity profiles, underscoring the importance of human-based models for accurate hazard assessment.

Recognition of these advantages has driven development of the DNT-IVB, formalized through OECD’s Initial Recommendations for evaluating DNT *in vitro* data in hazard assessment and weight-of-evidence determinations (OECD, 2023; Smirnova et al., 2024). Many DNT assays already meet test-readiness criteria for regulatory use (Bal-Price et al., 2018a; Bal-Price et al., 2018b) with practical validation demonstrating real-world utility. Klose et al. (2022) showed that DNT-IVB assays outperformed the EPA’s ToxCast high-throughput screening dataset for prioritizing flame retardants in regulatory risk assessment workflows, confirming the battery’s value in chemical prioritization and safety evaluation.

The transition to *in vitro* DNT models substantially advances replacement principles in chemical safety assessment, combining efficiency with human relevance while maintaining regulatory rigor.

## 3 Analysis of components in human cell-based DNT-IVB assays

Of the 17 DNT-IVB assays, seven utilize human cells differentiated into various neural subtypes, while others, such as the synaptogenesis assay and the neural network formation (NNF) assay, incorporate rat-derived cells (OECD, 2023). Initially, the NNF assay relied on rat cortical cells, but recent adaptations integrate human induced pluripotent stem cell (iPSC)-derived neurons (SynFire induced neurons), and primary human astroglia to improve human relevance and circumvent differences between species (Bartmann et al., 2023). Ongoing efforts are aimed at completely replacing rat cell-based assays with alternatives based on human cells (Tal et al., 2024).

### 3.1 Overview of human cell-based assays described within the DNT-IVB

The seven human cell-based assays of the DNT-IVB, analyzed in this review, are briefly introduced below, highlighting used cells, DNT endpoints and relevance (Table 3). They target key neurodevelopmental processes from neural progenitor proliferation to network formation, providing comprehensive coverage of DNT-relevant biology.

**TABLE 3:** Human cell-based assays of the DNT-IVB.

Assay name	Cells	DNT endpoints	Biological relevance	References
NPC1 (neural progenitor cell proliferation)	Human primary neural progenitor cells (hNPCs)	Proliferation, viability, cytotoxicity	Early neurodevelopment (NPC survival/proliferation under toxicant exposure)	Moors et al. (2009), Fritsche et al. (2011), Baumann et al. (2014), Baumann et al. (2016), Masjosthusmann et al. (2018), Nimtz et al. (2019), Masjosthusmann et al. (2020), Klose et al. (2022), Koch et al. (2022), Blum et al., 2023; OECD (2023)
NPC2-5 (neural progenitor cell migration and differentiation)	hNPCs differentiation to radial glia, neurons, oligodendrocytes	Migration distance, neurite length/area, neuronal differentiation, oligodendrocyte differentiation	Neural lineage development, structural integrity	Moors et al. (2009), Fritsche et al. (2011), Baumann et al. (2016), Dach et al. (2017), Schmuck et al. (2017), Masjosthusmann et al. (2018), Masjosthusmann et al. (2019), Masjosthusmann et al. (2020), Klose et al. (2022), Koch et al. (2022), Blum et al., 2023; OECD (2023)
UKN2 (cMINC)	Human iPSCs Differentiation to neural crest cells (NCCs)	NCC migration, viability	PNS development (sensory/autonomic/enteric neurons)	Zimmer et al. (2012), Zimmer et al. (2014), Pallocca et al. (2016), Nyffeler et al. (2017a), Nyffeler et al. (2017b), Nyffeler et al. (2018), Krebs et al. (2020), Blum et al., 2023; OECD (2023)
UKN4 (NeuriTox)	LUHMES differentiation to dopaminergic neurons	Neurite outgrowth,viability	CNS dopaminergic toxicity (Parkinson’s-relevant)	Lotharius et al. (2005), Scholz et al. (2011), Stiegler et al. (2011), Krug et al. (2013), Schildknecht et al. (2013), Scholz et al. (2013), Smirnova et al. (2016), Delp et al. (2018a), Delp et al. (2018b), Gutbier et al. (2018), Scholz et al. (2018), Brüll et al. (2020), Loser et al. (2021), Van der Stel et al. (2021), Blum et al., 2023; OECD (2023)
UKN5 (PeriTox)	Human iPSCs differentiation to dorsal root ganglia cells	Neurite outgrowth, viability, function	PNS neuron toxicity (pain/motor coordination)	Hoelting et al. (2016), Delp et al. (2018b), Holzer et al. (2022), Blum et al., 2023; OECD (2023), Holzer et al. (2025)
USEPA1 (high-content imaging assay)	Human iPSC-derived glutamatergic neurons	Neurite outgrowth	General neuritogenesis	Harrill et al. (2011), Druwe et al. (2016), Harrill et al. (2018), OECD (2023)
USEPA2 (cell viability, apoptosis and high-content imaging)	hNP1 (WA09-derived neuroprogenitor cells)	Proliferation,apoptosis, cytotoxicity	Cell death/proliferation interplay	Druwe et al. (2015), Carstens et al. (2022), OECD (2023)

The hNPC Proliferation Assay (NPC1) employs human primary neural progenitor cells (hNPCs) to assess the proliferation, viability, and cytotoxicity following test compound exposure (OECD, 2023, Appendix B.1) (Moors et al., 2009; Fritsche et al., 2011; Baumann et al., 2014; Baumann et al., 2016; Masjosthusmann et al., 2018; Nimtz et al., 2019; Masjosthusmann et al., 2020; Klose et al., 2022; Koch et al., 2022; Blum et al., 2023). This assay models early neurodevelopment by evaluating hNPC capacity to proliferate and survive toxicant exposure.

The hNPC migration and differentiation Assay (NPC2-5) involve both the maintenance and differentiation of hNPCs into specific neural lineages, such as neurons, radial glial cells, and oligodendrocytes (OECD, 2023, Appendix B.2) (Moors et al., 2009; Fritsche et al., 2011; Baumann et al., 2016; Dach et al., 2017; Schmuck et al., 2017; Masjosthusmann et al., 2018; Masjosthusmann et al., 2019; Nimtz et al., 2019; Masjosthusmann et al., 2020; Klose et al., 2022; Koch et al., 2022; Blum et al., 2023). The assay is designed to evaluate DNT-relevant endpoints, such as migration distance of neural progenitors, neurite length and area, neuronal differentiation (quantified by neuron number), and oligodendrocyte differentiation (measured by oligodendrocyte number). This assay offers a comprehensive evaluation of how test compounds affect neural lineage development and the structural integrity of the nervous system.

The cMINC Neural Crest Cell Migration Assay (UKN2) uses human iPSCs, differentiated into neural crest cells (NCCs), to assess migration and viability following exposure to test compounds (OECD, 2023, Appendix B.3) (Zimmer et al., 2012; Zimmer et al., 2014; Pallocca et al., 2016; Nyffeler et al., 2017a; Nyffeler et al., 2017b; Nyffeler et al., 2018; Krebs et al., 2020; Blum et al., 2023). This assay is valuable for studying the effects of chemicals on NCC function, which is crucial for the development of the peripheral nervous system. It evaluates the ability of NCCs to migrate and survive under chemical exposure, offering insights into potential developmental disruptions in sensory, autonomic, and enteric neurons.

The NeuriTox Neurite Outgrowth of CNS (central nervous system) Neurons Test (UKN4), also known as the NeuriTox Test, involves the differentiation of LUHMES cells, a human neuronal cell line, into dopaminergic neurons to assess toxicity within the dopaminergic system (OECD, 2023, Appendix B.4) (Lotharius et al., 2005; Scholz et al., 2011; Stiegler et al., 2011; Krug et al., 2013; Schildknecht et al., 2013; Scholz et al., 2013; Smirnova et al., 2016; Delp et al., 2018a; Delp et al., 2018b; Gutbier et al., 2018; Scholz et al., 2018; Brüll et al., 2020; Loser et al., 2021; Van der Stel et al., 2021; Blum et al., 2023). The assay specifically investigates how test compounds affect neurite outgrowth and cell viability in dopaminergic neurons. By focusing on dopaminergic neurons, this assay contributes to understanding the long-term effects of chemicals on late-onset diseases such as Parkinson’s disease.

The PeriTox Neurite Outgrowth of peripheral nervous system (PNS) Neurons Test (UKN5) also focuses on the PNS by differentiating human iPSCs into dorsal root ganglia (iDRG) neurons (OECD, 2023, Appendix B.5) (Hoelting et al., 2016; Delp et al., 2018b; Holzer et al., 2022; Blum et al., 2023; Holzer et al., 2025). This assay evaluates the viability and functionality of peripheral neurons following exposure to test compounds. It is especially valuable for investigating potential damage to peripheral nerve function, including pain perception and motor coordination.

The High-Content Imaging Assay screens for changes in neurite outgrowth due to chemical exposure in human iPSC-derived glutamatergic neurons (OECD, 2023, Appendix B.9) (Harrill et al., 2011; Druwe et al., 2016; Harrill et al., 2018). Neurite outgrowth serves as a sensitive indicator of DNT. For readability, this assay is referred to as USEPA1 in the following sections.

The Cell Viability, Apoptosis, and High-Content Imaging Assay evaluates neuroprogenitor cell (hNP1, WA09-derived) proliferation, apoptosis, and cytotoxicity (OECD, 2023, Appendix B.10) (Druwe et al., 2015; Carstens et al., 2022). The assay elucidates complex interplay between cell death pathways and proliferation inhibition. For readability, this assay is referred to as USEPA2 in the following sections.

These assays were systematically analyzed with respect to their reliance on animal-derived components and the availability of animal-free, xeno-free, and chemically defined alternatives (Table 4). The references to presented protocol details can be found in Table 4.

**TABLE 4:** Analysis of the use of animal-free, xeno-free and chemically defined components in the human cell-based DNT-IVB assays.

Assay	Used cell types	ECM/Coating		Medium		Critical media components		Other critical components		Literature
NPC1	Human primary neural progenitor cells (hNPCs, #PT-2599, Lonza)	Poly-(2-hydroxyethyl methacrylate) (poly-Hema, #P3932, Sigma-Aldrich)	Synthetic	Dulbecco’s modified Eagle medium (DMEM), high glucose (#31966, Gibco)	Animal-free, chemically defined	B27 (#175040, Gibco)	Contains bovine serum albumin (BSA), human recombinant insulin, human transferrin	StemPro Accutase (#A11105, Gibco)	Free of mammalian products, derived from crustaceae	Masjosthusmann et al. (2020), Klose et al. (2021), Klose et al. (2022), Koch et al. (2022), Blum et al., 2023; OECD (2023), Koch et al. (2025), Kühne et al. (2026)
​	​	​	​	Hams F-12 (#31765, Gibco)	Animal-free, chemically defined	Epidermal growth factor (EGF, #PHG0313, Gibco)	Human recombinant, reconstituted in Dulbecco’s phosphate-buffered saline (DPBS)	​	​	
​	​	​	​	​	​	Fibroblast growth factor (FGF) basic (#233-FB, R&D Systems)	Human recombinant; reconstituted in 0.1% or 1% BSA (#11920, Serva)	​	​	
NPC2-5 (Maintenance)	hNPCs (#PT-2599, Lonza)	Poly-Hema (#P3932, Sigma-Aldrich)	Synthetic	DMEM, high glucose (#319660, Gibco)	Animal-free, chemically defined	B27 (#175040, Gibco)	Contains BSA, human recombinant insulin, human transferrin	​	​	Masjosthusmann et al. (2020), Klose et al. (2021), Klose et al. (2022), Koch et al. (2022), Blum et al., 2023; OECD (2023), Koch et al. (2025), Kühne et al. (2026)
​	​	​	​	Hams F-12 (#31765, Gibco)	Animal-free, chemically defined	EGF (#PHG0313, Gibco)	Human recombinant, reconstituted in Dulbecco’s phosphate-buffered saline (DPBS)	​	​	
​	​	​	​	​	​	FGF basic (#233-FB, R&D Systems)	Human recombinant; reconstituted in 0.1% or 1% BSA (#11920, Serva)	​	​	
NPC2-5 (Differentiation)	HNPCs (#PT-2599, Lonza)	Poly-D-lysine (PDL, #P0899, Sigma-Aldrich)	Synthetic	DMEM, high glucose (#319660, Gibco)	Animal-free, chemically-defined	N-2 supplement (#175020, Gibco)	Animal-free, chemically defined	Anti-O4 IgM mouse (#MAB1326, R&D Systems)	Monoclonal, mouse-derived hybridoma cell line	​
​	​	Laminin (#L2020, Sigma-Aldrich)	Engelbreth-Holm-Swarm (EHS) sarcoma-derived	Hams F12 (#31765, Gibco)	Animal-free, chemically defined	EGF (#PHG0313, Gibco)	Human recombinant, reconstituted in Dulbecco’s phosphate-buffered saline (DPBS)	Anti-mouse Alexa 488 IgM (#A21042, Invitrogen)	Polyclonal, goat-derived	
​	​	​	​	​	​	FGF basic (#233-FB, R&D Systems)	Human recombinant; reconstituted in 0.1% or 1% BSA (#11920, Serva)	Anti-ßIII-Tubulin 647 IgG rabbit (#ab190575, abcam)	Monoclonal, rabbit recombinant	
​	​	​	​	​	​	​	​	Goat serum (#G9023, Sigma-Aldrich)	Goat-derived	
UKN2 (Maintenance)	Human induced pluripotent stem cells (hiPSCs, IMR90_clone_#4, WiCell)	Laminin-521 (#LN521, BioLamina) or human laminin (Sigma-Aldrich)	Human recombinant	Essential 8 (E8) (#A15170, Gibco)	Animal-free, chemically defined	​	​	​	​	Masjosthusmann et al. (2020), OECD (2023); Magel et al. (2024)
UKN2 (prior to differentiation on Matrigel)	HiPSCs differentiated to neural crest cells (NCCs)	Matrigel (#354234, Corning)	EHS-derived	E8 (#A15170, Gibco)	Animal-free, chemically defined	​	​	​	​	
UKN2 (Differentiation on Matrigel)	HiPSCs differentiated to neural crest cells (NCCs)	Matrigel (#354234, Corning)	EHS-derived	KnockOut DMEM (#10829018, Gibco)	Contains BSA, insulin transferrin	KnockOut Serum Replacement (KSR) (#108280; Gibco)	Contains BSA	​	​	
​	​	​	​	​	​	Noggin (#719-NG, R&D Systems)	recombinant murine, reconstituted in 0.1% BSA	​	​	​
​	​	​	​	DMEM/F-12 (#21331, Gibco)	Animal-free, chemically defined	Apo-transferrin (#T2036, Sigma-Aldrich)	Human origin	​	​	
​	​	​	​	​	​	Insulin (#I9278, Sigma-Aldrich)	Human recombinant	​	​	
​	​	​	​	​	​	Progesterone (#P7556, Sigma-Aldrich)	Synthetic	​	​	
​	​	​	​	​	​	Putrescine (#P5780, Sigma-Aldrich)	Synthetic	​	​	
UKN2 (Differentiation on PLO/laminin/fibronectin)	HiPSCs differentiated to NCCs	Poly-L-ornithine (PLO, #P3655, Sigma-Aldrich)	Synthetic	DMEM/F-12 (#21331, Gibco)	Animal-free, chemically defined	Apo-transferrin (#T2036, Sigma-Aldrich)	Human origin	​	​	
​	​	Laminin (#L2020, Sigma-Aldrich) or human laminin (Sigma-Aldrich)	EHS-derived	​	​	Insulin (#I9278, Sigma-Aldrich)	Human recombinant	​	​	
​	​	Fibronectin (#F1141, Sigma-Aldrich)	Bovine serum-derived	​	​	Progesterone (#P7556, Sigma-Aldrich)	Synthetic	​	​	
​	​	​	​	​	​	Putrescine (#P5780, Sigma-Aldrich)	Synthetic	​	​	
​	​	​	​	​	​	selenium (#S5261, Sigma-Aldrich)	Synthetic	​	​	
​	​	​	​	​	​	EGF (#236-EG, R&D Systems)	Human recombinant; reconstituted in 0.1% BSA	​	​	​
​	​	​	​	​	​	FGF basic (#233-FB, R&D Systems)	Human recombinant; reconstituted in 0.1% BSA	​	​	
UKN4 (Maintenance)	LUHMES cells	PLO (#P3655, Sigma-Aldrich)	Synthetic	Advanced DMEM/F-12 (#126340, Gibco)	Contains BSA, insulin transferrin	N-2 supplement (#175020, Gibco)	Animal-free, chemically defined	Trypsin (#25300, Gibco)	Porcine pancreas-derived	(Masjosthusmann et al., 2020; OECD, (2023) M. Leist, personal communication (January, 2025))
​	​	Fibronectin (#F1141, Sigma-Aldrich)	Bovine serum-derived	​	​	FGF basic (#4114-TC, R&D Systems)	Human recombinant; reconstituted in 0.1% BSA	​	​	
UKN4 (Differentiation)	LUHMES cells differentiated to dopaminergic neurons	PLO (#P3655, Sigma Aldrich)	synthetic	Advanced DMEM/F-12 (#126340, Gibco)	Contains BSA, insulin transferrin	glial cell line-derived neurotrophic factor (GDNF, #212-GD, R&D Systems)	Recombinant human, reconstituted in 0.1% BSA	​	​	
​	​	Fibronectin (#F1141, Sigma Aldrich)	Bovine serum-derived	​	​	Dibutyryl-cAMP (#D0627, Sigma-Aldrich)	Synthetic	​	​	
UKN5 (Maintenance)	hiPSCs EPTHELIAL-1 (#IPSC0028, Sigma-Aldrich)	Laminin-521 (#LN521, BioLamina)	Human recombinant	E8 (#A15170, Gibco)	Animal-free, chemically defined	​	​	​	​	Masjosthusmann et al. (2020), Holzer et al. (2022); OECD (2023), Holzer et al. (2025)
UKN5 (prior to differentiation on Matrigel)	HiPSCs	Matrigel (#354234, Corning)	EHS-derived	E8 (#A1517001, Gibco)	Animal-free, chemically defined	​	​	​	​	
UKN5 (differentiation)	HiPSCs differentiated to dorsal root ganglia (iDRG)	Matrigel (#354234, Corning)	EHS-derived	KnockOut DMEM (#10829018, Gibco)	Contains BSA, insulin transferrin	KSR (#108280; Gibco)	Contains BSA	​	​	
​	​	​	​	Advanced DMEM/F-12 (#126340, Gibco)	Contains BSA, insulin transferrin	Noggin (#719-NG, R&D Systems)	recombinant murine, reconstituted in 0.1% BSA	Cryopreservation in FBS (#A15-101, PAA)-containing medium	Contains FBS	
​	​	​	​	​	​	Apo-transferrin (#T2036, Sigma-Aldrich)	Human origin	​	​	
​	​	​	​	​	​	Insulin (#I9278, Sigma-Aldrich)	Human recombinant	​	​	
​	​	​	​	​	​	Progesterone (#P7556, Sigma-Aldrich)	Synthetic	​	​	
​	​	​	​	​	​	Putrescine (#P5780, Sigma-Aldrich)	Synthetic	​	​	
​	​	​	​	​	​	selenium (#S5261, Sigma-Aldrich)	Synthetic	​	​	
USEPA1	Human iPSC-derived glutamatergic-enriched cortical neurons (iCell GlutaNeurons, #R1061, FujiFilm Cellular Dynamics, CDI)	PLO (#P3655, Sigma-Aldrich)	Synthetic	BrainPhys medium (#05790, StemCell Technologies)	Animal-free, chemically defined	ICell Neural Supplement B (#M1029, CDI)	Chemically defined with trace amounts of purified human protein	anti-β-Tubulin III antibody (#802001, Biolegend)	Polyclonal, rabbit-derived	(Harril et al. (2011); Druwe et al. (2016); Harril et al. (2018); OECD, (2023)), T. Shafer, personal communication, (February 2025)
​	​	Laminin (#L2020, Sigma-Aldrich)	EHS-derived	​	​	ICell Nervous System Supplement (#M1031, CDI)	Chemically defined with recombinant human growth factors	Goat anti-rabbit Alexa 546 IgG (#A11010, Invitrogen)	Polyclonal, goat-derived	
​	​	​	​	​	​	N-2 supplement (#17502, Gibco)	Animal-free, chemically defined	Blocking and antibody solution	Contain BSA	
USEPA2	Human neural progenitor cell line hNP1 (ArunA Biomedical)	PLO (#P3655, Sigma-Aldrich)	Synthetic	KnockOut DMEM/F-12 (#12660012, Gibco)	Contains BSA, insulin transferrin	StemPro Neural Supplement (#A1050801, Gibco)	Animal-free, recombinant	TrypLE Express (#12604013, Gibco)	Animal-free, recombinant	(Druwe et al., 2015; Harrill et al., 2018; OECD, 2023), T. Shafer, personal communication, February 2025)
​	​	Laminin (#L2020, Sigma-Aldrich)	EHS-derived	​	​	EGF (#PHG0314, Gibco)	Recombinant human, reconstituted in phosphate buffered saline (PBS)	Cryopreservation in proliferation medium with 10% DMSO	Contains BSA, insulin transferrin	
​	​	​	​	​	​	FGF basic (#PHG0024, Gibco)	Recombinant human, reconstituted in water	​	​	

### 3.2 Analysis of the use of animal-derived or animal-free, xeno-free and chemically defined components in the human cell-based assays of the DNT-IVB

This review systematically examines the materials employed in cell culture and downstream analyses within these seven assays (Table 4). Emphasis is placed on the often-overlooked presence of animal-derived components in protocols and commercial formulations, including BSA, ECM preparations such as Matrigel and animal-sourced antibodies.

These animal-derived reagents, and also human blood-derived products, are frequently associated with undefined or incompletely characterized composition and batch-to-batch variability, which can compromise reproducibility and limit translational relevance (Aisenbrey and Murphy, 2020; Cassotta et al., 2022; Nezvedova et al., 2025; Wolff and Hendrix, 2025; Akuta et al., 2026).

For instance, Matrigel and other tumor- or tissue-derived ECM preparations exhibit pronounced batch-to-batch and even within-batch variability in biochemical and mechanical properties, hindering standardization of 3D and organoid cultures (Hughes et al., 2010; Aisenbrey and Murphy, 2020). Likewise, animal-generated antibodies and serum-derived albumin supplements can introduce poorly characterized, batch-variable backgrounds that affect assay performance and data interpretation (Gray et al., 2020; DeLuca et al., 2021; Duarte et al., 2023; Nezvedova et al., 2025; Akuta et al., 2026).

The increasing availability of recombinant and chemically defined reagents provides opportunities to reduce reliance on animal-derived materials and to improve the definition of cell culture systems used in toxicological testing (Nesterenko et al., 2020; Cassotta et al., 2022; Fraser et al., 2025). Sequence-defined or recombinant products can improve traceability and manufacturing consistency relative to reagents purified directly from animal tissues or biological fluids, which are often chemically undefined (Bradbury and Plückthun, 2015).

However, variability is not unique to animal-derived materials, and animal-free reagents do not inherently guarantee reproducible behavior. HSA purified from blood can contain a certain percentage of undefined components (Rafnsdóttir et al., 2023). Recombinant proteins, including growth factors, may also exhibit batch-to-batch variability arising from differences in expression systems, host cell biology, post-translational modifications or manufacturing conditions (O'Flaherty et al., 2020; Wang et al., 2025). In addition, even synthetic additives and plastics can contribute to variability. For instance, impurities in the surfactant Poloxamer 188 have been shown to induce cytostatic effects and atypical cell culture performance during monoclonal antibody production (Bandyopadhyay et al., 2022), while inter-laboratory studies in cell-free systems demonstrate that reagent preparation and site-specific factors can substantially influence experimental variability (Cole et al., 2019).

Moreover, replacing major components, such as serum, ECM preparations or metabolic activation systems may alter assay biology. Changes in baseline phenotype, sensitivity, or readouts, have been reported even when technical adaptation is successful (Perez-Diaz et al., 2023; Reichstein et al., 2023; Fraser et al., 2025).

Taken together, these observations indicate that while animal-free reagents can support the development of more defined *in vitro* systems, their adoption alone does not automatically reduce variability. Rigorous validation, careful quality control, and transparent reporting of cell culture materials remain essential.

#### 3.2.1 Cell culture media components

##### 3.2.1.1 Basal cell culture media and cryopreservation media

Basal media such as Dulbecco’s Modified Eagle Medium (DMEM), Ham’s F-12 (F-12), or DMEM/F-12 combinations are inherently xeno-free, protein-free, and chemically defined (Eagle, 1959; Ham, 1965). All seven analyzed DNT-IVB assays have successfully eliminated FBS, establishing serum-free protocols across stem cell or NPC maintenance and neural differentiation phases (Table 4; Figure 2A).

Chemically defined maintenance media include E8 (UKN2/5), DMEM and F-12 (NPC1/NPC2-5), DMEM/F-12 (UKN2) and BrainPhys medium (USEPA1) (Bardy et al., 2015). Despite elimination of FBS, BSA often remains prevalent in cell culture media for nutrient transport and cellular stabilization (Francis, 2010; Price, 2017; Belinskaia et al., 2021). Such serum-free but BSA-containing formulations are Advanced DMEM/F-12 including AlbuMAXII (UKN4/5), Knockout DMEM containing AlbuMAX I (UKN2), and Knockout DMEM/F-12 containing AlbuMAX I (USEPA2) (Paul et al., 1998; Nam, 2015).

Recombinant human serum albumin (rHSA) has advantages in conformational stability and reduced risk of adventitious impurities as it is not isolated from mammalian blood (Mishra and Heath, 2021; Wynendaele et al., 2021). Cost barriers seem to be minimal: at typical 0.1% supplementation, rHSA (€8.69/g protein) versus BSA (€4.33/g protein) yields only €0.0022 difference per 5 mL medium, if applied with 0.1% (v/v; Bio&Sell, Germany, pricing for 6 March 2026).

##### 3.2.1.2 Cell culture media supplements

Cell culture supplements provide essential nutrients and growth factors for serum-free cell culture that support cell survival, proliferation, and differentiation (Romijn et al., 1984; Romijn, 1988). The analyzed DNT-IVB assays predominantly rely on BSA-containing supplements (Table 4; Figure 2B). B27, a popular supplement in stem cell culture and neural differentiation protocols, used in NPC1 and NPC 2-5, contains BSA as indicated in the product information (Brewer and Cotman, 1989; Brewer et al., 1993). It supports neuronal viability when combined with Neurobasal Medium, by providing essential nutrients and growth factors (Brewer and Cotman, 1989; Brewer et al., 1993). Knockout Serum Replacement (KSR) used in UKN2, UKN5, includes BSA as well (AlbuMAX I) (Paul et al., 1998).

Xeno-free alternatives exist but vary in definition status. N-2 supplement, used in NPC2-5, UKN4, USEPA1, is xeno-free and chemically defined, containing insulin, transferrin, progesterone, putrescine, and selenium (Bottenstein & Sato, 1979). iCell Nervous System Supplement, used in USEPA1, applies only recombinant human growth factors, achieving full chemical definition, whereas iCell Neuronal Supplement B, used in USEPA1, contains traces of purified human protein despite being labeled as “chemically defined” (A. Othman, FCDI, personal communication, March 2025).

Current “serum-free” labeling does not include distinctions between animal-free, xeno-free and chemical definition status, often requiring patent analysis for complete composition. Tiered labeling (serum-free, xeno-free and chemically defined) would facilitate informed reagent selection to develop defined assays.

Manufacturers and assay developers can collaboratively advance this transition. Reformulation substituting BSA with rHSA offers potential to reduce quality variability while maintaining functionality. Assay developers can either initiate new protocols with rHSA or validate equivalence in established assays. Concurrently, increased market demand for rHSA-containing supplements could drive price competitiveness, facilitating broader adoption in cell culture.

##### 3.2.1.3 Growth factors

Growth factors and signaling proteins drive stem cell differentiation and neural linage commitment across the analyzed DNT-IVB assays (Table 4). The assays employ human recombinant growth factors: epidermal growth factor (EGF) is used in NPC1, NPC 2-5 and UKN2, fibroblast growth factor (FGF) and FGF basic (FGF2) are used in NPC2-5, UKN2, UKN4 and USEPA2 and glial cell line-derived neurotrophic factor (GDNF) is used in UKN4. However, minimal BSA quantities (0.1%–1%) remain as carrier protein in growth factor formulations all assays except USEPA2, which reconstitutes EGF in phosphate-buffered saline (PBS) and FGF2 in water, avoiding the use of BSA.

Noggin is essential for neural lineage induction via bone morphogenic protein (BMP) antagonism (Chambers et al., 2009). UKN2/UKN5 assays use murine recombinant Noggin reconstituted in 0.1% BSA (Masjosthusmann et al., 2020). Human and murine Noggin exhibit high sequence homology and functional equivalence (Holley and Ferguson, 1997; Pauklin and Vallier, 2015), with comparable pricing (recombinant human Noggin, €9.54/µg; recombinant murine Noggin, €10.93/µg, R&D Systems, Bio-Techne GmbH, Germany, pricing for 6 March 2026). Current use of recombinant murine Noggin is scientifically justified given proven efficacy and biological conservation. For new assay development, recombinant human Noggin would further reduce xenogenic components and enhance consistency with human biology.

#### 3.2.2 ECM components

**FIGURE 1:**
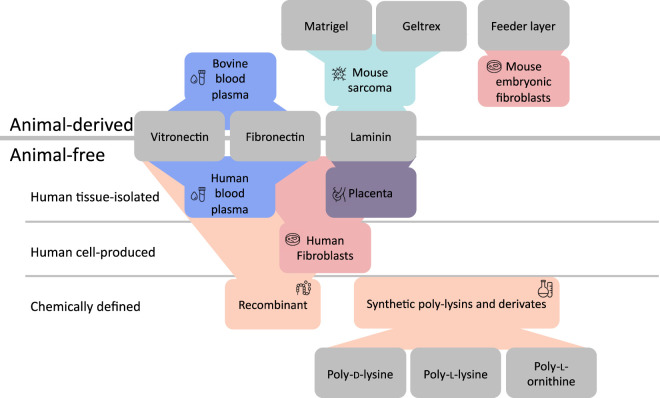
Overview of extracellular matrix (ECM) components used in the human cell-based assays of the DNT-IVB and commercially available alternatives, categorized by their origin. Animal-derived components, such as murine Engelbreth-Holm-Swarm (EHS) sarcoma-derived Matrigel, Geltrex, or mouse embryonic fibroblast feeder layers, are commonly used but raise ethical concerns and issues related to variability. To reduce animal dependency, intermediate solutions for glycoprotein production, including vitronectin, fibronectin, and laminin, are sourced from both bovine blood and human tissue isolation, as well as through chemically defined recombinant protein production. Additionally, synthetic homopolypeptides can be incorporated into cell culture systems to enhance reproducibility.

Neuronal cells, particularly those derived from stem cells, require a supportive ECM environment that mimics their natural niche to maintain their viability, morphology, and function, which is why additional ECM components are used as coating during cultivation to enhance cell adhesion, growth, and differentiation (Friedl and Brocker, 2000; Theocharis et al., 2016; Nicolas et al., 2020). ECM glycoproteins, like laminin, fibronectin, vitronectin or collagen, mediate stem cell adhesion, pluripotency maintenance, lineage commitment, migration, and neural differentiation through integrin signaling and biomechanical cues (Watt and Huck, 2013; Theocharis et al., 2016; Hagbard et al., 2018). Figure 1 illustrates commercial source variability across animals, human tissue-derived, and recombinant production systems.

Animal-derived matrices closely recapitulate native complexity. Matrigel, a murine Engelbreth-Holm-Swarm (EHS) sarcoma extract, supports iPSC pluripotency maintenance, neural induction, and multi-linage differentiation through synergistic laminin, collagen IV, entactin, perlecan, and growth factor blends (Kleinman et al., 1986; Kleinman and Martin, 2005). This multi-component bioactivity facilitates neurite outgrowth, synapse formation, and network maturation, key DNT endpoints, explaining its widespread adoption despite known batch-to-batch variability (Kleinman et al., 1986; Flanagan et al., 2006; Ma et al., 2008; Hughes et al., 2010; Li et al., 2014; Long and Huttner, 2019).

**FIGURE 2:**
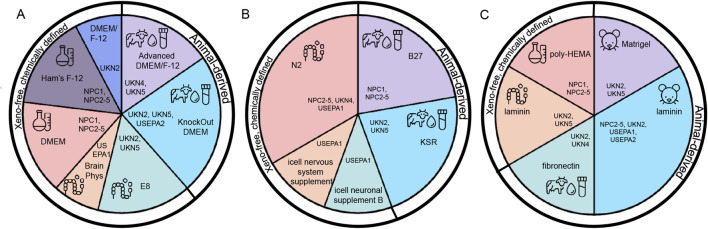
Classification of reagents used in human-cell based DNT-IVB assays according to their origin. **(A)** Basal cell culture media categorized as animal-derived or chemically defined and xeno-free. **(B)** Cell culture supplements classified by their inclusion of animal-derived components (e.g., B27, KSR, containing bovine serum albumin (BSA) versus xeno-free alternatives (e.g., N2, iCell Nervous System Supplement). **(C)** Extracellular matrix (ECM) components and surface coatings are divided into animal-derived products (e.g., Matrigel, murine laminin), recombinant products (laminin) or synthetical coating (poly-HEMA), which in this case prevents cell attachment.

Accordingly, the DNT-IVB assays reflect a strong reliance on animal-derived ECM components (Table 4; Figure 2C). All seven analyzed DNT-IVB human cell-based assays, except non-adherent NPC1/NPC2-5 (poly-HEMA), use such materials: Matrigel is used for iPSC neural induction and differentiation in UKN2 and UKN5, EHS-derived laminin supports neuronal maturation, as in NPC2-5, UKN2, USEPA1 and USEPA2, and bovine plasma-derived fibronectin facilitates cell attachment in UKN2 and UKN4. Recombinant human laminin (e.g., LN521) is currently limited to iPSC maintenance in UKN2 and UKN5.

Despite their functional advantages, animal-derived matrices introduce significant limitations, including batch-to-batch compositional and mechanical variability and undefined growth factor content, which can confound lineage commitment and reproducibility (Hughes et al., 2010). In addition, high numbers of animals are needed for their production, with approximately 16 mice required per 100 mL Matrigel (Kibbey, 1994; Kleinman, 2001; Berg and Kurreck, 2021).

To address these limitations, more defined ECM alternatives have been developed, including the use of single glycoproteins such as laminin, fibronectin, and vitronectin, which promote integrin-mediated adhesion (Hayashi and Furue, 2016; Aisenbrey and Murphy, 2020; Kawase and Nakatsuji, 2023). As illustrated in Figure 1, ECM glycoproteins are derived from diverse sources with distinct limitations. Laminin, an often-used alternative to Matrigel, can be extracted from murine EHS sarcoma (Timpl et al., 1979), while fibronectin and vitronectin are commonly isolated from bovine blood plasma (Engvall and Ruoslahti, 1977). All three glycoproteins can also be obtained from human tissue, being xeno-free, but still entails batch-to-batch variability. Consequently, only the recombinant versions provide fully chemically defined alternatives.

Nevertheless, the transition to defined alternatives remains challenging. Recombinant ECM are often associated with substantially higher costs (e.g., LN521: €622/mg vs. EHS laminin: €296/mg, Biolamina, Sweden and Sigma-Aldrich, Germany, pricing for 6 March 2026). More importantly, single-component systems frequently fail to replicate the biochemical and biophysical complexity of native EHS-derived matrices. This reduction in complexity can alter cell behavior, affecting adhesion, morphology, and differentiation efficiency, and may require extensive protocol re-optimization across all stages from stem cell maintenance to neuronal maturation.

To partially address these limitations, combinatorial approaches, for example, laminin with poly-L-lysine or poly-L-ornithine, are used to enhance cell adhesion through electrostatic interactions and better approximate ECM functionality (Liu et al., 2020; Hartmann et al., 2023). Other strategies include synthetic or recombinant matrices, such as peptide-functionalized polyethylene glycol (PEG) hydrogels or engineered protein polymers (e.g., ZT^Fn^), which allow precise control over biochemical composition and mechanical properties. Such animal-free matrices have demonstrated applicability in defined experimental settings. For example, Schwartz et al. (2015) generated highly uniform hPSC-derived neural constructs on PEG hydrogels, supporting controlled pluripotency exit, neural induction, and toxicity-responsive neurogenesis. Similarly, ZT^Fn^, a human protein based recombinant polymer modified with fibronectin domains, supports pluripotency maintenance and neural crest differentiation (Hill et al., 2019; Nesterenko et al., 2019; Nesterenko et al., 2020). Laminin E8 fragments have also been shown to sustain iPSC pluripotency and enhance cell adhesion compared to Matrigel (Miyazaki et al., 2012). In a proof-of-concept study, Nesterenko et al. (2020) demonstrated that ZT^Fn^ can substitute bovine serum-derived fibronectin as a coating for the UKN2 assay, yielding comparable bioactivity and sensitivity to model toxicants.

However, current evidence is largely based on proof-of-concept studies, and systematic comparisons between animal-derived and fully defined alternatives across DNT-IVB endpoints remain scarce. Notably, potential limitations such as reduced cell adhesion or altered differentiation efficiency are likely underreported, as negative or non-optimizing results are rarely published. This creates a knowledge gap that complicates the objective assessment of replacement strategies.

Resources such as the Basement Membrane Extract (BME)-free database (RRID:SCR_026058) can support the identification of animal-free hydrogels and coatings tailored to specific cell types (3Rs Centre Utrecht, 2025). While these tools facilitate the identification of alternatives, further standardization and head-to-head comparison studies are required to enable broader adoption.

#### 3.2.3 Other components

##### 3.2.3.1 Antibodies

DNT-IVB immunofluorescence staining employs antibodies across production technologies for neural and glial lineage identification (Table 4). NPC2-5 assays combine mouse hybridoma-derived anti-O4 monoclonal for oligodendrocyte detection with a goat anti-mouse polyclonal Alexa 488 IgM secondary. An anti-β-Tubulin III recombinant monoclonal rabbit Alexa 647 antibody is used in NPC2-5. The USEPA1 assay employs a rabbit polyclonal anti-β-Tubulin III IgG primary antibody, detected by a goat-derived polyclonal anti-rabbit Alexa 546 IgG secondary antibody.

Hybridoma monoclonals like anti-O4 require initial mouse immunization for hybridoma establishment, which is associated with animal welfare considerations, particularly in protocols involving repeated immunization or ascites production (Miri et al., 2026). Once cell lines exist, production does not require further animal experiments, though sequence recovery enables full recombinant transition (Gray et al., 2020). Beyond animal use, hybridoma technology presents additional limitations, including relatively long development times when animals must be immunized, compared to *in vitro* phage display technologies (Laustsen et al., 2021), the potential presence of multiple immunoglobulin-producing genes within a single clone (Bradbury et al., 2018), and susceptibility to genetic drift during long-term culture, which may affect reproducibility (Miri et al., 2026). Accordingly, recommendations support continued use of well-characterized existing hybridomas but discourage the generation of new ones in favor of animal-free approaches such as phage display (Laustsen et al., 2021; Viegas Barroso et al., 2020; Gray et al., 2020).

Recombinant primaries (anti-βIII-tubulin NPC2-5) leverage phage display technology from synthetic libraries, eliminating immunization entirely while providing CHO/HEK scalability and batch consistency (Winter et al., 1994). However, polyclonal secondaries perpetuate animal dependence through serum immunization, offering signal amplification via multi-epitope binding but introducing batch variability (Kahn et al., 2024).

This hybridoma-to-recombinant progression in NPC2-5 assays mirrors broader trends toward animal-free antibody production that eliminates batch variability while enabling molecular engineering opportunities such as affinity maturation and multi-formatting (humanization, Fc-modifications) (Bradbury and Plückthun, 2015; Bradbury et al., 2021). Initial cost and availability gaps between recombinant and traditional antibodies continue to narrow as research demand drives manufacturing scale-up (Gray et al., 2020). Also, secondary antibodies, currently the primary animal dependence in DNT-IVB immunofluorescence staining, tend to follow this trajectory as commercial recombinant alternatives mature. However, recombinant secondary antibodies remain less extensively validated in practice, requiring additional validation efforts before full substitution can be achieved (Kahn et al., 2024), mirroring the media/supplement evolution observed earlier.

Progress toward animal-free immunofluorescence continues, aligning with regulatory recommendations (Viegas Barroso et al., 2020) and batch consistency needs for reproducible DNT assessment *in vitro*.

##### 3.2.3.2 Reagents used in immunofluorescence staining

The NPC2-5 and USEPA1 immunofluorescence stainings employ animal-derived components: In the NPC2-5 assays, the blocking solution combines 50% goat serum with 5% BSA, while antibody solutions contain 10% goat serum and 1% BSA. In the USEPA1 assay 2% BSA is used in both blocking/permeabilization buffers and 0.5% in antibody solutions.

Animal-free blocking alternatives offer comprehensive solutions: plant-derived blockers (e.g., Vector Laboratories Animal-Free Blocker) eliminate immunoglobulin cross-reactivity while detergent-based formulations (Tween-20, polyvinylpyrrolidone) minimize protein adsorption and maintain optimal signal-to-noise ratios (Haycock, 1993). Recent work has also demonstrated the practical feasibility of animal-free staining workflows using recombinant antibodies and a plant-derived Animal-Free Blocker solution (Miri et al., 2026).

While transition requires revalidation, these alternatives offer batch consistency benefits, as animal-derived sera exhibit documented batch-to-batch contamination risks that can compromise experimental outcome.

##### 3.2.3.3 Reagents used for subcultivation

NPC1 assay employs crustacean-derived Accutase for neural progenitor neurosphere dissociation and BrdU proliferation quantification, representing a mammalian-free but non-chemically defined enzyme. In UKN4 porcine pancreas-derived trypsin is traditionally used for cell handling. Although widely used, this enzyme introduces potential variability due to its animal origin and carries a risk of contamination with impurities such as chymotrypsin or other proteases. USEPA2 protocol for hNP1 cell subcultivation employs a recombinant fungal protease (TrypLE Express). TrypLE Express contains no animal components at any stage of production.

## 4 Relevance of animal component-free, xeno-free and chemically defined components in human cell-based assays for risk assessment and regulatory use

Human cell-based assays for regulatory chemical risk assessment increasingly prioritize animal-free, xeno-free and chemically defined components to potentially enhance protocol transparency, transferability and standardization. Regulatory frameworks already provide clear guidance in his direction: the OECD Good Ín Vitro Method Practices (GIVIMP) guidelines recommend minimizing or eliminating undefined components such as FBS (OECD, 2018), while the Centre for the Validation of Alternative Methods Scientific Advisory Committee (ESAC) recommends avoiding FBS use unless scientifically justified (van der Valk et al., 2018).

Chemically defined media are increasingly viewed as the gold standard for achieving maximal reproducibility in cell-based methods. However, fully defined formulations remain challenging to implement for many systems (Galbraith et al., 2018; Ritacco et al., 2018; van der Valk et al., 2018). In practice, serum-free media, even when not completely chemically defined, are often sufficient for high-throughput screening and hazard identification, where robustness and scalability outweigh complete compositional transparency (Perez-Diaz et al., 2023; Pfeifer et al., 2024). This also applies to the assays of the DNT-IVB, which have already demonstrated to fulfill readiness criteria to be fit-for-purpose for screening and prioritization and use within Integrated Approaches to Testing and Assessment (IATA) (Fritsche et al., 2017; Bal-Price et al., 2018a; Smirnova et al., 2024).

Significant progress has been achieved in replacing FBS in the field of neural cell culture. Nevertheless, animal-derived components persist, most notably BSA, which functions as a stabilizing agent and carrier protein. BSA is present in widely used supplements such as B27, KSR, Knockout DMEM, Knockout DMEM/F-12 (via AlbuMAXI) and Advanced DMEM/F-12 (via AlbuMAXII). Its biological origin introduces batch-to-batch variability (Hulse et al., 2013), highlighting opportunities for further refinement toward more defined and standardized systems.

ECM components represent a critical, yet comparatively under-addressed, source of variability. Complex matrices such as Matrigel remain widely used due to their robust support of cell attachment and differentiation, despite their undefined and animal-derived nature. The comparatively limited emphasis on ECM in guidance documents likely reflects the current lack of universally applicable, fully defined replacement systems that can replicate the complex biochemical and structural cues required for diverse cell types. GCCP for stem cell culture endorses the xeno-free mTESR1 stem cell medium, yet Stemcell Technologies’ product sheet still recommends pairing it with Matrigel (Eskes et al., 2017). Within the DNT-IVB context, readiness criteria emphasize the importance of identifying sources of variability, primarily targeting FBS. Expanding this perspective to include ECM, since its role in neurodevelopmental endpoints like neurite outgrowth and synaptogenesis warrants consideration, where batch consistency metrics (e.g., laminin isoforms, growth factor bioactivity) are essential (Bal-Price et al., 2018a; Long and Huttner, 2019).

Additional components, including antibodies, also contribute to overall assay performance. Policy recommendations, such as those from the European Union Reference Laboratory for Alternatives to Animal Testing (EURL ECVAM), encourage the transition toward animal-free antibody production where scientifically feasible (Viegas Barroso et al., 2020). The increasing availability of recombinant antibodies offers new opportunities to enhance consistency and traceability.

A growing portfolio of animal-free alternatives, including rHSA, animal-free ECM, recombinant antibodies, and animal-free blocking reagents, supports the continued evolution of cell-based assays. Their integration, however, typically requires careful validation, including (Fritsche et al., 2017; Bal-Price et al., 2018a; Galbraith et al., 2018; Ritacco et al., 2018; van der Valk et al., 2018; Perez-Diaz et al., 2023; Pfeifer et al., 2024; Smirnova et al., 2024) protocol adaption, optimization of reagent concentrations, and confirmation of performance equivalence. This process represents a shared effort across academia, industry, and regulatory stakeholders.

In this context, standardized classification frameworks, such as BioLamina’s tiered labels (“serum-free,” “xeno-free,” “chemically defined”) aligned with International Society for Cell and Gene Therapy (ISCT) guidelines, contribute to transparency while accommodating proprietary formulations. Building on existing readiness criteria by more explicitly considering ECM- and antibody-related parameters could further support harmonization efforts and facilitate the transition toward fully defined, animal-free systems—while maintaining the performance and reliability required for regulatory applications.

## 5 Conclusion

Human cell-based DNT assays already represent an advance that reduces animal use while improving human relevance in chemical risk assessment. Significant progress toward serum-free conditions exists, though opportunities remain to address BSA-containing supplements (e.g., B27), animal-derived ECM, and animal-derived antibodies using recombinant alternatives.

A pragmatic two-pronged strategy accelerates this transition: (1) New protocols can incorporate animal-free design principles from inception. (2) Established assays can evaluate component reduction where feasible, balancing validation requirements with practicality. Manufacturers can contribute through standardized labeling (“serum-free/xeno-free/chemically defined”) and expanded animal-free reagent availability. Updated GCCP/GIVIMP guidelines could explicitly address BSA and ECM as sources of variability alongside serum to support standardization.

This collaborative framework addresses the human cell-based assays of the DNT-IVB while offering transferable strategies for broader toxicological *in vitro* applications.

## Nomenclature

3Rs: replacement, reduction, refinement
B27: B27 supplement
BME: Basement Extract Membrane
BMP: bone morphogenic protein
BSA: bovine serum albumin
CNS: central nervous system
DMEM: Dulbecco’s Modified Eagle Medium
DMEM/F-12: DMEM/Ham’s F-12
DNT: developmental neurotoxicity
DNT-IVB: DNT *in vitro* Battery
DPBS: Dulbecco’s phosphate-buffered saline
E8: Essential 8 medium
ECM: extracellular matrix
EFSA: European Food Safety Authority
EGF: epidermal growth factor
EPA: US Environmental Protection Agency
EURL ECVAM: European Union Reference Laboratory for Alternatives to Animal Testing
FBS: fetal bovine serum
FGF: fibroblast growth factor
FGF2: fibroblast growth factor basic
GCCP: Good Cell Culture Practice
GDNF: glial cell line-derived neurotrophic factor
GIVMP: Good *in vitro* Method Practices
hNPC: primary human neural progenitor cells
hNP1: human neural progenitor cell line 1
rHSA: recombinant human serum albumin
iDRG: dorsal root ganglia cells derived from iPSCs
iPSC: induced pluripotent stem cell
ISCT: International Society for Cell and Gene Therapy
KSR: KnockOut Serum Replacement
LN521: laminin-521
LUHMES: Lund human mesencephalic neuroblastoma cell line
NAMs: New Approach Methodologies
NCC: neural crest cells
NNF: neural network formation
OECD: Organization for Economic Co-operation and Development
PNS: peripheral nervous system
TG: test guideline
**ZT** ^**FN**^: ZT-Fibronectin polymer
